# Terahertz thermal curve analysis for label-free identification of pathogens

**DOI:** 10.1038/s41467-022-31137-2

**Published:** 2022-06-16

**Authors:** S. W. Jun, Y. H. Ahn

**Affiliations:** 1 Department of Physics, Ajou University, Suwon, 16499 Korea; 2 Department of Energy Systems Research, Ajou University, Suwon, 16499 Korea

**Keywords:** Imaging and sensing, Sensors and probes, Biophotonics, Metamaterials, Terahertz optics

## Abstract

In this study, we perform a thermal curve analysis with terahertz (THz) metamaterials to develop a label-free identification tool for pathogens such as bacteria and yeasts. The resonant frequency of the metasensor coated with a bacterial layer changes as a function of temperature; this provides a unique fingerprint specific to the individual microbial species without the use of fluorescent dyes and antibodies. Differential thermal curves obtained from the temperature-dependent resonance exhibit the peaks consistent with bacterial phases, such as growth, thermal inactivation, DNA denaturation, and cell wall destruction. In addition, we can distinguish gram-negative bacteria from gram-positive bacteria which show strong peaks in the temperature range of cell wall destruction. Finally, we perform THz melting curve analysis on the mixture of bacterial species in which the pathogenic bacteria are successfully distinguished from each other, which is essential for practical clinical and environmental applications such as in blood culture.

## Abstract (web summary)

A label-free sensing method has been developed for identifying hazardous pathogens based on their intrinsic properties. This was possible by interrogating the temperature-dependent dielectric constant of the microbes in the far-infrared range.

## Introduction

In the era of global pandemics, demand for the development of rapid and accurate tools is increasing for the identification of pathogens such as bacteria and viruses for the effective treatment of deadly diseases and the prevention of infections^1,2^. Polymerase chain reaction (PCR) techniques have been widely adopted for their identification, as it can detect various infectious bacteria with high fidelity^3–6^. Nevertheless, PCR is generally labor intensive and time consuming owing to its multiple procedures; additionally, it requires target-specific agents and pretreatment to extract DNA from microorganisms^7–10^. By contrast, alternative microbial detection techniques such as fluorescence microscopy and flow cytometry require fluorescent materials to efficiently detect microorganisms^11–14^. Therefore, true label-free diagnostic tools that exploit their inherent physical properties without requiring fluorescent labeling should be developed.

Recently, terahertz (THz) metamaterials have been introduced as a real-time and sensitive platform for detecting biological substances including microorganisms^15–20^. Because metamaterial sensing is dielectric sensing, obtaining information regarding the dielectric constant of the target substances is the primary step for the practical application of the sensors^21–27^. Recently, we discovered that microorganisms can be classified in terms of their dielectric properties; in other words, the dielectric constants of bacteria are higher than those of molds, whereas yeasts exhibit particularly high values that are greater than that of water^28^. Their differences were successfully interpreted in terms of their cell wall composition. Nevertheless, the identification of the individual species based on their dielectric properties is difficult because their dielectric indices are similar among different types of microorganisms.

More importantly, the peak shift of a metamaterial and its plasmonic resonance depend not only on its dielectric constants, but also on the number of microbes deposited around the gap area^15,16^. In addition, microbes do not have a spectral fingerprint in the THz frequency range, thereby limiting the capability of label-free identification of the microbes. Specific identification can be realized by functionalizing devices with an antibody specific to the target analytes^29^. However, this requires additional coating processes with expensive antibodies that are not sustainable in general, thereby resulting in sensors being disposed of^30,31^. Therefore, a novel approach for differentiating microbial species in terms of their intrinsic properties will enable a breakthrough in the development of early identification tools for pathogens.

In this study, we developed a label-free THz spectroscopic tool, in which we monitored the dynamic change in the refractive index of the microorganisms coated on the metamaterials while varying the substrate temperature. We obtained differential thermal curves by monitoring the temperature-dependent frequency shift of the metamaterial resonance, which offers unique fingerprints specific to individual microbes. We obtained the thermal curves for pathogenic microbes, whose peaks were consistent with the phase transition during the growth and death of the individual cells. In addition, we successfully decomposed the thermal curves of a mixture of pathogens into those of individual microbes.

## Results and discussion

**Fig. 1:**
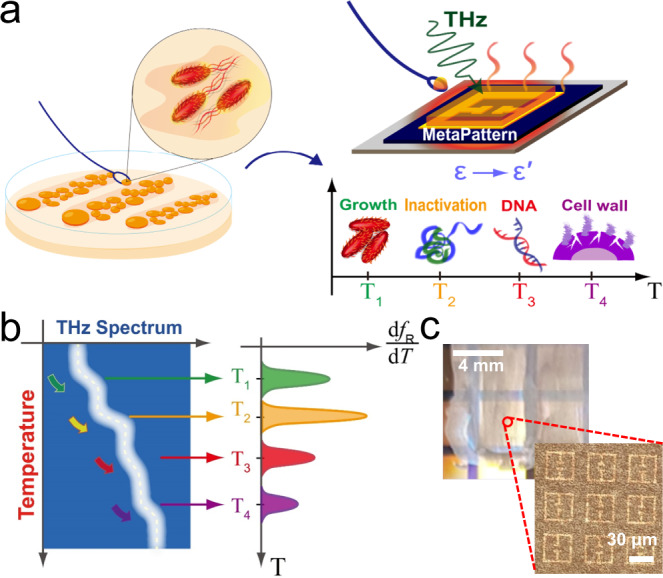
Schematic of thermal curve analysis based on THz metamaterials. **a** Schematic of experiments. The microbial films grown on a culture medium are transferred to THz metamaterials while samples are being heated. The microbes exhibit phase change with increasing temperature based on conditions for growth, inactivation, DNA denaturation, and cell wall destruction. **b** An abrupt change in the metamaterial resonance occurs in the transition between different growth and death phases, in which strong peaks appear in different thermal curves (DTCs). DTCs offer unique fingerprints for identifying different microbial species. **c** Photograph of metamaterial sensors coated with a microbial film (30-µm-thick *E. coli* film).

We developed a label-free identification tool for individual pathogens, such as bacteria, by performing THz thermal curve analysis, as schematically illustrated in Fig. 1a. We monitored the resonant frequency (*f*_R_) of THz metamaterials coated with a bacterial layer while changing the sample temperature from 25 to 160 °C. In general, cells experience multiple phases with increasing temperature, such as growth, thermal inactivation, DNA denaturation, and cell wall destruction^32–34^. In our experiments, we discovered a significant change in the THz dielectric constant at the transition temperature between the multiple stages of their growth and death phases, which varied significantly for different species. By measuring the metamaterial resonances at different temperatures, we can identify the individual microbial species, as schematically shown in Fig. 1b, without performing pretreatments such as labeling and DNA extraction. In particular, we obtained a differential thermal curve (DTC) from in situ THz spectroscopy, in which we discovered multiple peaks in the transition temperature; this enables us to identify the microbes even when they are mixed with other species.

As shown in Fig. 1c, the bacterial layer (*E. coli*, in this case) that was grown on the culture media was transferred onto the metasensors using a plastic loop. The details of the metasensor design are depicted in the Methods section; we used the arrays of split-ring resonators that deliver a high sensitivity reaching 80 GHz/RIU^35^. The thickness of the microbial layer was 20–30 μm which is larger than the saturation thickness considering the vertical extent of the effective sensing volume of our metasensors^35^. In this configuration, the amount of frequency shift will be governed by a couple of bacterial layers near the substrate; hence, the effect of the temperature gradient (away from the substrate) will be negligible. The bacterial biofilm contained extracellular polymeric substances (EPSs), which caused the cells to adhere to the substrate during the experiments. This is contrary to our previous study pertaining to bacterial films for dielectric constant measurements, in which we fabricated a thick pallet (100–300 μm) via a drying process^28^.

**Fig. 2:**
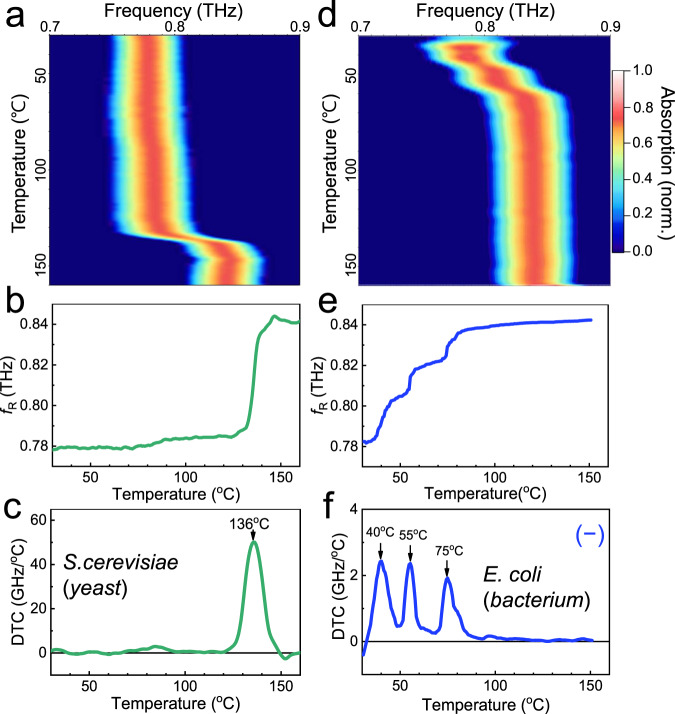
Experimental results for thermal curve analysis obtained from in situ THz spectroscopy. **a** 2D plot of THz absorption through metamaterials coated with a yeast layer (*S. cerevisiae*) as functions of the spectrum (*x*-axis) and temperature (*y*-axis). **b** Metamaterial resonance (*f*_R_) as a function of temperature extracted from **a**. **c** DTC (d*f*_R_/d*T*) for the yeast layer obtained by differentiating the curve in (**b**). **d** 2D plot of THz absorption for a metasensor coated with a *E. coli* layer. **e** *f*_R_–*T* plot extracted from **d**. **f** DTC for the *E. coli* layer. Minus sign (–) in **f** indicates that *E. coli* is the Gram-negative bacteria.

Figure 2 shows the results of the melting curve analysis based on in situ THz spectroscopy, where we gradually increased the temperature of the metasensors coated with bacterial and yeast layers. Figure 2a shows a two-dimensional (2D) plot of THz absorption as a function of frequency (*x*-axis) and temperature (*y*-axis) when the metasensor was coated with a 30-μm-thick yeast layer. Initially, the peak frequency was 0.77 THz at room temperature, which corresponds to the resonant frequency of the metasensor when covered by the dielectric films with an index of *ε*_film_ = 4.5 for the case involving the yeast layers^15^. In other words, the resonant frequency indicated a red-shift (from the original value of 0.87 THz) after the coating was introduced owing to the change in the dielectric configuration. By contrast, *f*_R_ increased as the dielectric constant of the coated film decreased with temperature. As the temperature increased, *f*_R_ did not change significantly until it reached 136 °C, and a significant blue-shift to 0.84 THz was observed. This indicates that the dielectric constant of the yeast layer decreased at the abovementioned temperature, as mentioned previously. We also note that the metamaterial resonance did not change noticeably in the absence of the microbial films over the temperature range we tested (Supplementary Fig. 1). By fitting the curve, we extracted a plot of *f*_R_ as a function of temperature *T*, as shown in Fig. 2b. This corresponds to the dielectric constant of the film changing from *ε*_film_ = 4.5 to 2.1, which can be extracted from the amount of frequency shift as discussed elsewhere (Supplementary Fig. 2)^28^.

As shown in Fig. 2c, the change in the resonant frequency is more clearly depicted based on the differential thermal curve, i.e., based on d*f*_R_/d*T*. As mentioned earlier, the most prominent peak appeared at 136 °C, when the yeast exhibited a significant change in the dielectric index. In fact, this is the temperature at which polysaccharides (i.e., *β*-glucans) in yeast cells are dissociated^36^. Because the dielectric constant of the yeasts is governed by that of the cell wall composition (i.e., *β*-glucans with long polymeric chain length), which has a high THz dielectric index^28^, the temperature-dependent change in the film index is dominated by the cell wall destruction process. The dielectric constants of the microorganisms as a function of temperature have not been previously addressed in THz and other frequency ranges. Because the characteristics of the temperature-dependent change in the dielectric index vary for different species, the THz thermal curves offer unique fingerprints for the rapid identification of bacteria without being affected by the amount of coated substances on the metasensors.

More intriguing phenomena with pronounced peaks were discovered for the bacterial species. Figure 2d–f shows the in situ THz spectroscopy results for obtaining the thermal curve of the *E. coli* bacterium, which is one of the most important pathogens that serves as an indicator of bacteriological quality in water. The 2D plots of absorption and the *f*_R_–*T* plot are shown in Fig. 2d, e, respectively. The dielectric constant of the *E. coli* layer was as high as that of the yeast layer when it contained EPSs; in fact, it was higher than that of a dried *E. coli* film (Supplementary Fig. 3). The relative contribution of the EPS to the thermal curve is yet to be elucidated. However, it is noteworthy that water comprises most of the constituents of the EPS in the microbial layer (reaching 97%)^37^, and the microbial substances in the EPS are those of intracellular components such as polysaccharides, structural proteins, enzymes, nucleic acids, lipids, and other compounds^38^. We extracted differential thermal curves for *E. coli* (Fig. 2f), which exhibited a behavior distinct from that of yeast. In other words, the DTC peaks appeared at 40, 55, and 75 °C, which corresponded to growth, thermal inactivation, and DNA denaturation, respectively^34,39,40^. Contrary to the yeast film, the *E. coli* layer did not exhibit noticeable peaks in the higher temperature range (130–140 °C). This is likely because the bacterial cell wall composition is peptidoglycan with a relatively low dielectric index of 1.8^28^, and more importantly, Gram-negative bacteria such as *E. coli* have relatively thin walls (1.5–10 nm) compared with Gram-positive bacteria (20–80 nm) and yeast (70 nm), which will be discussed in more detail later.

The decrease in the dielectric constant (i.e., the blue shift in the metasensor resonance) with increasing temperature can be explained based on cell expansion and the molecular structural changes occurring during their growth and death processes. For instance, it is well known that at the growth temperature, the cell undergoes proteolysis process in which bacterial enzymes break proteins into their component amino acids^34,41–44^. Conversely, the thermal inactivation processes are primarily due to the denaturation of proteins; a decrease in the DC dielectric constant at high temperatures has been reported previously^45^. Furthermore, it is implied that DNA denaturation decreases the dielectric indexes^46^; nonetheless, the detailed characteristics in the THz range must be further addressed. Importantly, the heat-induced changes in their morphology cannot be identified through the optical microscope, whereas the morphology change according to the cell wall destruction could be recognized by scanning electron microscopy (Supplementary Fig. 4). In addition, thermal gravimetric analysis on the microbial films reveals that temperature-dependent mass changes do not influence our DTC results noticeably, because there is a gradual mass loss owing to water evaporation (Supplementary Fig. 5)^47^. In that regard, the THz dielectric information of the microbial films will work as an effective indicator for monitoring their dynamical phases in the wide range of microbiology procedures. For instance, we could address the cell dynamics by performing the in situ THz spectroscopy for monitoring their growth and death at a specific temperature (Supplementary Fig. 6).

**Fig. 3:**
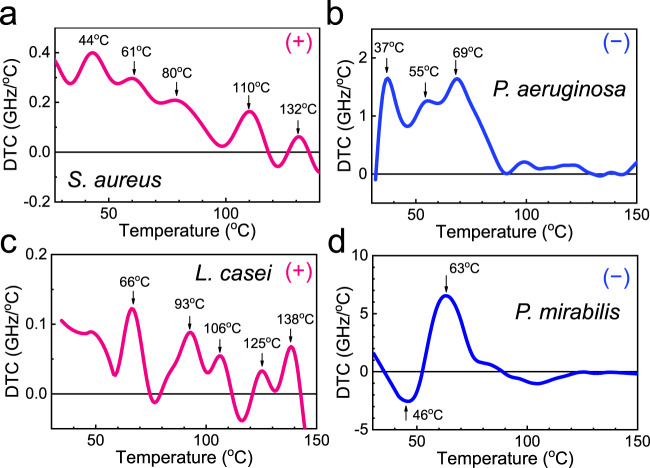
Differential thermal curve results for four bacterial species. DTCs for **a** *S. aureus*, **b** *P. aeruginosa*, **c** *L. casei*, and **d** *P. mirabilis*. Gram-negative bacteria (*P. aeruginosa* and *P. mirabilis*) do not exhibit noticeable peak corresponding to the cell wall destruction (i.e., at *T* > 100 °C). Positive (+) and negative (–) signs denote the Gram-positive and Gram-negative bacteria, respectively.

We obtained thermal curves for other bacterial species, including critical pathogens that cause severe sepsis. An example of the thermal curves for four different bacteria is illustrated in Fig. 3. *S. aureus* (Gram-positive type), whose DTC is shown in Fig. 3a, is a pathogen that typically causes skin and respiratory infections^48^. *P. aeruginosa* (Gram-negative type), shown in Fig. 3b, causes inflammation and fatal sepsis in the lungs^49^. Meanwhile, *L. casei* (Gram-positive type) is a probiotic bacterium, whose thermal curve is shown in Fig. 3c. Finally, as shown in Fig. 3d, the thermal curve of *P. mirabilis* (Gram-negative type) is widely distributed in soil and water in the natural environment and causes 90% of proteus infections in humans^50^.

The DTCs for different bacteria differed significantly from each other, particularly in terms of their peak positions. These peaks represent the specific temperatures during the transition between the multiple phases during their growth and death. For instance, *S. aureus* grows rapidly at 44 °C, is thermally inactivated at 61 °C, and is denaturized (of DNA) at 80 °C, and our DTC peak values are consistent with the abovementioned values. Conversely, the peaks at 110 and 132 °C (which do not appear in the *E. coli* case) are likely associated with cell wall destruction because the temperature for the dissociation of the cell wall components (such as peptidoglycan) was measured at >100 °C, although it has not been reported specifically for *S. aureus*^51,52^. Notably, the DTC peaks corresponding to the cell wall destruction appeared for *S. aureus* (Fig. 3a) and *L. casei* (Fig. 3c), which are Gram-positive bacteria, whereas they did not appear for Gram-negative bacteria such as *P. aeruginosa* (Fig. 3b), *P. mirabilis* (Fig. 3d), and *E. coli* (Fig. 2f).

It is noteworthy that some microorganisms exhibit peaks superimposed on a broad background, e.g., *S. aureus* (Fig. 3a). This is likely due to an overall temperature-dependent change in the microbial films, e.g., the gradual removal of the EPS or the thermal expansion of the film. Interestingly, the DTC for *P. mirabilis* showed a negative peak at 46 °C, which indicates that the dielectric index increased around the growth temperature. This is contrary to the observation for typical bacteria and is likely due to their unique swarming characteristics that tend to increase the density of the microbial film during the growth phase^53^.

**Fig. 4:**
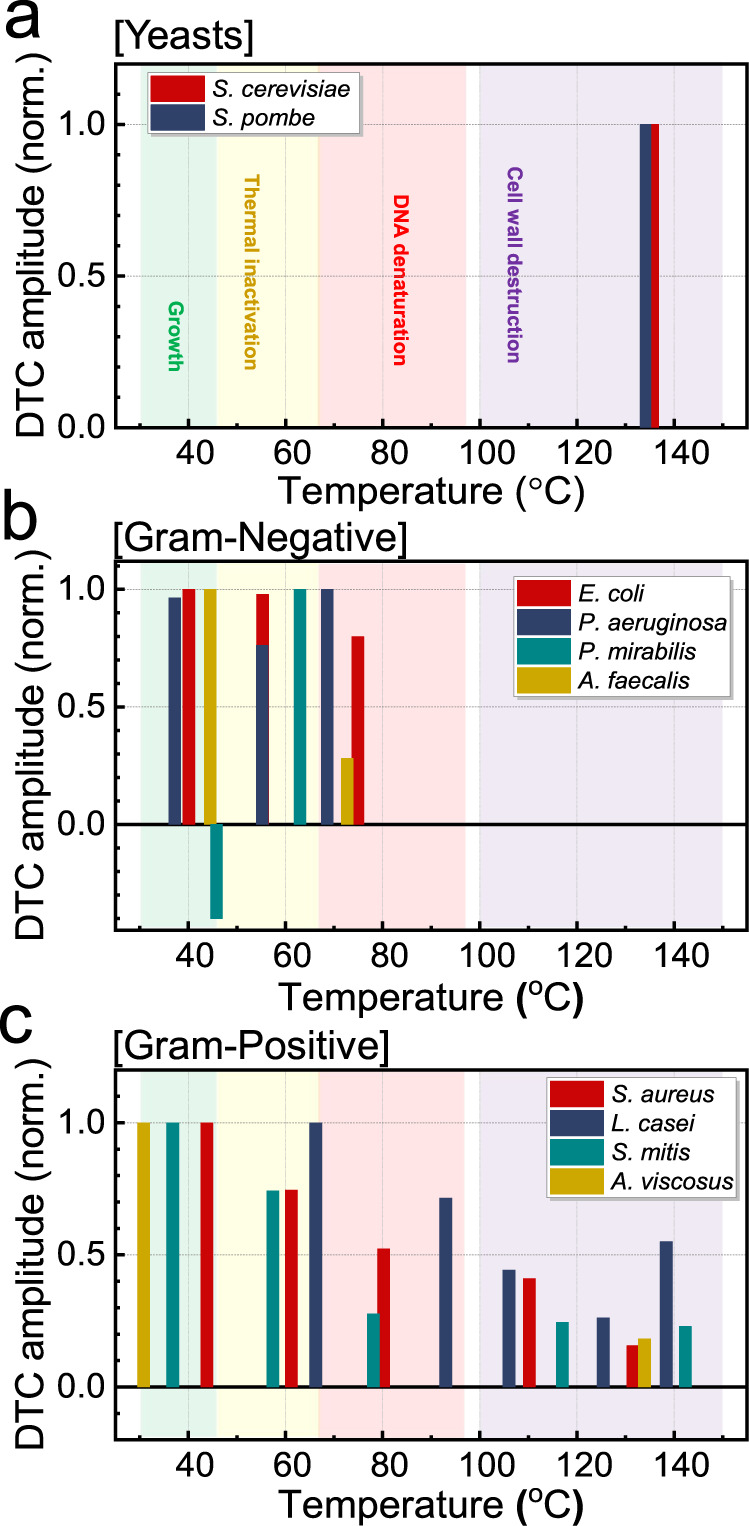
Summary on the thermal curve analysis for 10 pathogens. Bar graphs of amplitude as a function of temperature according to the peaks observed in DTCs for **a** yeasts, **b** Gram-negative bacteria, and **c** Gram-positive bacteria. The amplitudes are normalized by its highest peak values for each species.

The results of the DTC analysis are summarized in Fig. 4 for the yeasts (two species) and bacteria (eight species) in terms of the peak amplitudes vs. temperature plots (Supplementary Figs. 7–8 and Supplementary Table 1). We present the DTC for the yeasts (*S. cerevisiae* and *S. Pombe*) in Fig. 4a, whereas we categorized bacteria into Gram-negative (Fig. 4b) and Gram-positive (Fig. 4c) types. All of them exhibited multiple peaks that can serve as unique fingerprints for their identification. Here, the peaks at the temperature bands shaded by green (30–45 °C), yellow (46–66 °C), red (68–95 °C), and purple (100–150 °C) indicate the temperature ranges for the growth, thermal inactivation, DNA denaturation, and cell wall destruction, respectively, as described above. We discovered that many peak positions were consistent with those reported in the literature (Supplementary Table 1), which validates our approach. They were determined via various biological approaches, including optical density measurement, flask methods, PCR, and differential scanning calorimetry methods; however, conventional methods are typically time consuming or require significant amounts of specimens^8,9,32,33^. More importantly, although the dielectric constants of the bacteria are similar, our melting curve analysis extracted from the temperature-dependent dielectric constants enables their identification according to the species. It is noteworthy that the peak positions in the melting curves can deviate from our data for different growth conditions in the culture medium; it has been frequently reported that their temperature-dependent characteristics change according to the growth conditions and the thickness of the layer (Supplementary Figs. 9–10)^34^.

More importantly, our technique proved to be extremely effective in discriminating the bacteria type, i.e., whether they are Gram-positive and Gram-negative bacteria, as implied in Figs. 2 and 3. In other words, strong peaks in the purple range (corresponding to the cell-wall destruction) appeared only in the Gram-positive case (Fig. 4c), whereas no peak was observed in the Gram-negative case (Fig. 4b)^54^. In other words, cell wall destruction imposed a more prominent effect on the temperature-dependent dielectric indexes of the Gram-positive bacteria than those of the Gram-negative bacteria. Gram staining has been widely adopted to distinguish and classify bacterial species into two groups; however, gram staining requires labeling, and distinction in terms of cell wall composition has not been investigated sufficiently. Therefore, our technique is promising for the early identification of bacteria type and will particularly benefit the early diagnosis of sepsis^55,56^.

**Fig. 5:**
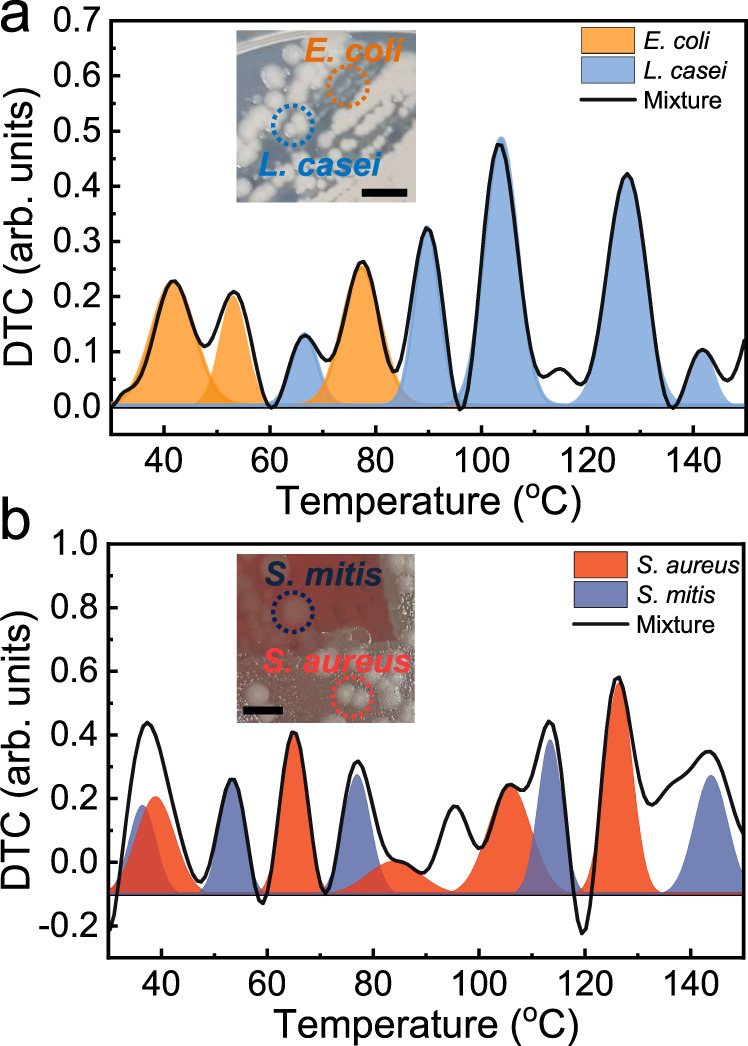
Thermal curve analysis for a mixture of bacteria. **a** DTC for a mixture of *E. coli* and *L. casei* bacteria. The curve was decomposed by those of *E. coli* (orange) and *L. casei* (sky-blue) based on their respective peak positions obtained from Fig. 4. **b** DTC for a mixture of *S. aureus* and *S. mitis* bacteria, which was decomposed by those of *S. aureus* (red) and *S. mitis* (blue). Insets in (**a**, **b**) are the pictures of bacterial layers grown in respective culture media (scale bar: 5 mm).

Finally, we performed a THz thermal curve analysis on a mixture of bacterial species. The label-free detection of infectious agents and the discrimination of non-pathogenic bacteria from pathogenic bacteria is essential for practical applications such as blood culture. As shown in Fig. 5a, we tested a mixture comprising *E. coli* and *L. casei*, which are prevalent in the human gut. We cultivated them simultaneously using the same culture media of nutrient agar, which is typically used for testing a wide range of bacteria regardless of their type. The inset shows a photograph of the two bacteria grown in the culture medium before they were transferred to the metasensors. In the thermal curve analysis, we discovered multiple peaks, and by fitting the curves, we were able to decompose them into those of *E. coli* (orange) and *L. casei* (sky-blue). In other words, it was possible to discriminate *E. coli*, which tends to be hazardous depending on their amount and location, among relatively favorable microbes such as Lactobacillus. In addition, this technique may be extremely useful when examining the relative amount of Lactobacillus to *E. coli* in the human gut, which is an important indicator of human health^57–59^. In addition, as shown in the inset of Fig. 5a, they were not clearly distinguished from each other in terms of color and shape; therefore, they could not be clearly differentiated even under a microscope^60^.

Another example is shown in Fig. 5b, in which a DTC for a mixture comprising *S. aureus* and *S. mitis* is presented; both bacteria inhabit the upper tract of humans and often cause severe human diseases. We cultivated the two bacterial species using the respective culture media (i.e., nutrient agar for *S. aureus*, and blood agar for *S. mitis*), as shown in the photograph of the inset (captured before they were transferred to the metasensors). Similarly, multiple peaks were observed, which corresponded to those of *S. aureus* and *S. mitis*. This further confirms the effectiveness of our technique in identifying specific pathogens that share habitats. In addition, we observed some peaks that did not correspond to the individual species, as shown in Fig. 5a, b; this should be investigated further. More importantly, our method allows us to identify pathogens in the early stage based on their intrinsic properties without necessitating procedures such as fluorescent labeling as well as antibodies. We will be able to improve the sensitivity further, for instance, by confining the pathogens in the gap area (Supplementary Figs. 11)^61^. In future studies, on-site label-free sensors with which microbes can be identified the soonest possible with enhanced sensitivity for single-cell identification should be investigated.

In summary, we developed label-free sensors for identifying pathogens based on thermal analysis using THz metamaterials. The resonant frequency of the metasensors coated with the bacterial layer changed as a function of temperature, in accordance with the temperature-dependent dielectric constants of the layer. We obtained DTCs from the in situ measurement of the resonant frequency as a function of temperature, which provided a unique fingerprint specific to the individual bacterial species without the use of fluorescent dyes and antibodies. We obtained the thermal curves for 10 pathogens that cause deadly human diseases, such as sepsis. The peaks in the DTCs were consistent with the temperature-dependent bacterial phases such as growth, thermal inactivation, DNA denaturation, and cell wall destruction. More importantly, we discovered that the Gram-negative bacteria did not exhibit noticeable peaks owing to cell wall destruction, which was contrary to the positive type. This has a great clinical importance enabling their early discrimination without the need for gram stain tests. Finally, we performed a THz melting curve analysis on a mixture of bacterial species, in which the pathogenic bacteria were successfully distinguished from the non-pathogenic ones. This is in fact essential for practical clinical and environmental applications such as blood culture.

## Methods

### Preparation of microorganism samples

We prepared microbial layers comprising representative bacteria and yeasts. They were grown via streaking on a medium, followed by incubation for 2 days. The microorganisms were obtained either from the Korean Agricultural Culture Collection (KACC) or the Korean Collection for Type Cultures (KCTC). The culture medium and the incubation temperature varied by sample as follows: *Escherichia coli* (KACC 11598; nutrient agar; 37 °C), *Proteus mirabilis* (KCTC 2510; nutrient agar; 37 °C), *Streptococcus mitis* (KCTC 5650; blood agar; 37 °C), *Actinomyces viscosus* (KCTC 9146; blood agar; 37 °C), *Alcaligenes faecalis* (KCTC 2678; nutrient agar; 37 °C), *Pseudomonas aeruginosa* (KCTC 1750; nutrient agar; 37 °C), *Lactobacillus casei* (KCTC 13086; MRS agar; 37 °C), and *Staphylococcus aureus* (KCTC 1928; nutrient agar; 37 °C) for bacteria; *Saccharomyces cerevisiae* (KCTC 27139; glucose-peptone-yeast extract agar; 25 °C) and *Schizosaccharomyces pombe* (KCTC 27259; glucose-peptone-yeast extract agar; 25 °C) for yeasts.

### Fabrication of metamaterial devices

Metamaterial patterns (i.e., metasensors) were prepared using a conventional photolithography method on a high-resistivity Si substrate (resistivity > 3000 Ω cm and thickness of 550 μm). A metal film (Cr/Au, 3/97 nm) was deposited using a thermal evaporator to define the arrays of split-ring resonators with outer dimensions of 36 μm × 36 μm and a gap size of 3 μm. The linewidth was 4 μm. We transferred the microbial films grown on the culture media to the metasensors via a plastic inoculation loop with a film thickness of 10–30 μm.

### In situ THz time-domain spectroscopy

The real-time THz transmission amplitudes of the metamaterials with bacteria were measured using a conventional THz time-domain spectroscopy setup^62^. A femtosecond laser (with a pulse width of 30 fs at 800 nm) incident on the photoconductive antenna that emitted a linearly polarized THz pulse was focused on the metamaterials with a 1 mm spot diameter in a nitrogen-purged environment. The time traces of the transmitted THz electric field, both in amplitude and phase, were measured by varying the time delay between the 800 nm probe beam and the THz pulse. The THz spectrum was obtained by applying a fast Fourier transform to the time trace and normalized with respect to the reference. We monitored the transient THz absorption of metamaterials coated with bacteria while adjusting the temperature of the sample using a ceramic heater. The ceramic heater was punctured at the center with a diameter of 2 mm to enable the transmission experiments. We increased the temperature gradually from 25 to 160 °C for 40 min.

### Statistics and reproducibility

We obtained consistent DTCs for the repeated measurements for each species; importantly, the DTC peak positions were consistent with the known phase change temperatures (Supplementary Table 1). Future investigation with a full statistical analysis is required to address the effect of physiological variations of the microbes.

### Reporting summary

Further information on research design is available in the Nature Research Reporting Summary linked to this article.

## Supplementary information

Supplementary Information

Reporting Summary

## Data Availability

The data generated and analyzed during this study are available from the corresponding author upon reasonable request.
