# Supplementary material for: Terahertz thermal curve analysis for label-free identification of pathogens
Source: Nat Commun. 2022 Jun 16;13:3470. doi: 10.1038/s41467-022-31137-2 (PMC9203813; doi:10.1038/s41467-022-31137-2)
Supplement: Supplementary file 1 — Supplementary Information [file 41467_2022_31137_MOESM1_ESM.pdf]

Supplementary Information

Terahertz Thermal Curve Analysis for Label-free Identification of Pathogens

S. W. Jun and Y. H. Ahn[*]

Department of Physics and Department of Energy Systems Research, Ajou University,

Suwon 16499, Korea

[*]Electronic mail: ahny@ajou.ac.kr

S1. Temperature dependent metamaterial resonance

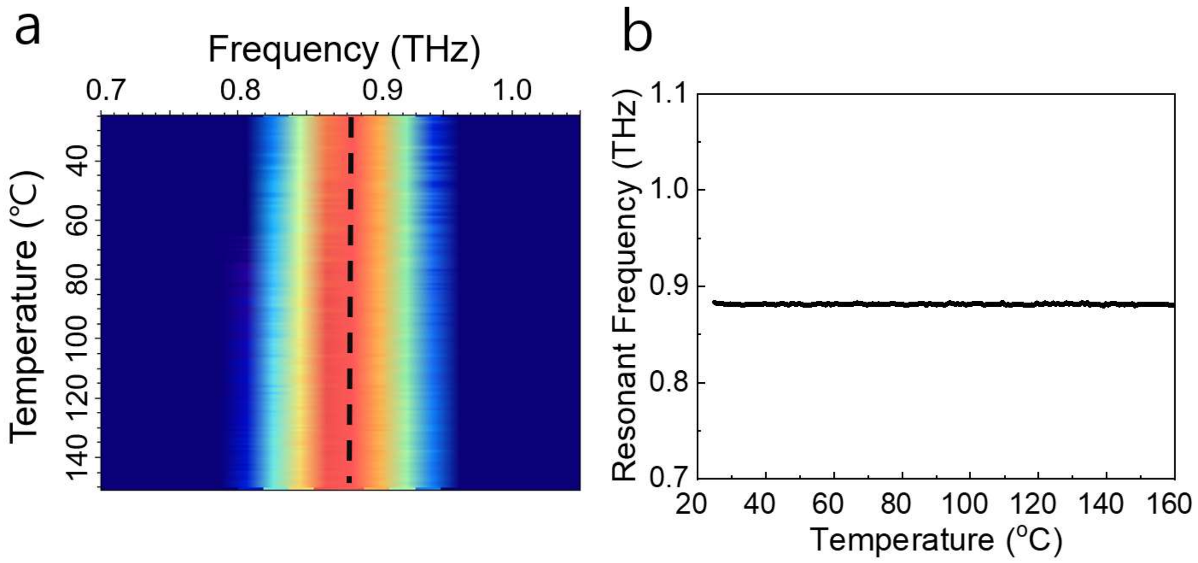

Supplementary Figure 1 a 2D plot of THz absorption through metamaterials as functions of spectrum (*x*-axis) and temperature (*y*-axis) without the coating of microbial layers. **b** Resonant peak position extracted from **a**, which confirms that there's no noticeably change in the resonant frequency as a function of temperature.

S2. Temperature dependent dielectric constant of yeast film

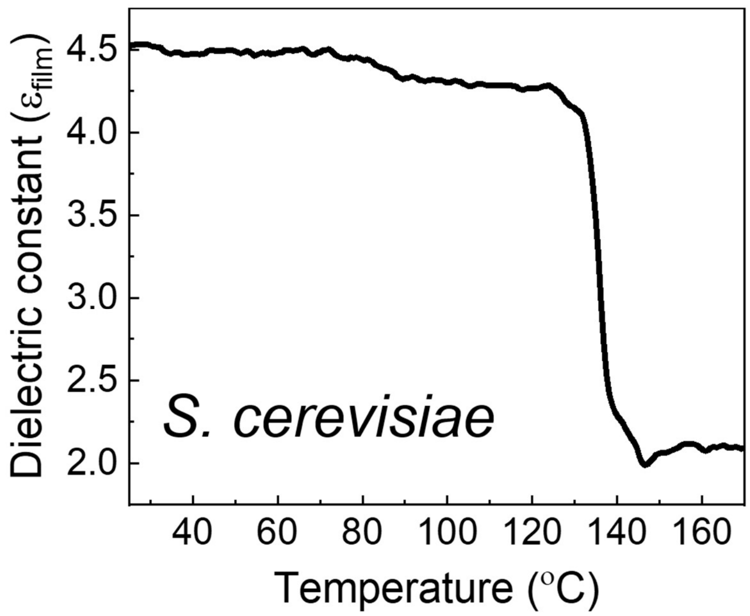

Supplementary Figure 2 The dielectric constant (ε_{film}) was obtained from the frequency shift (Δf) of the metasensor (as shown in Fig. 2b of the main text). We used the explicit relation of $\Delta f/f_0 = \alpha(\varepsilon_{film} - \varepsilon_{air})/\varepsilon_{eff}$, with the resonant frequency of $f_0 = 0.87$ THz, the effective substrate index of ε_{eff} =6.32 (determined by the combination of the substrate and air indexes), and the coefficient α of 0.1886[1,2]. This relation holds on the condition that the film thickness is larger than that of saturation (~20 μm).

S3. Dielectric constant of *E. coli* film

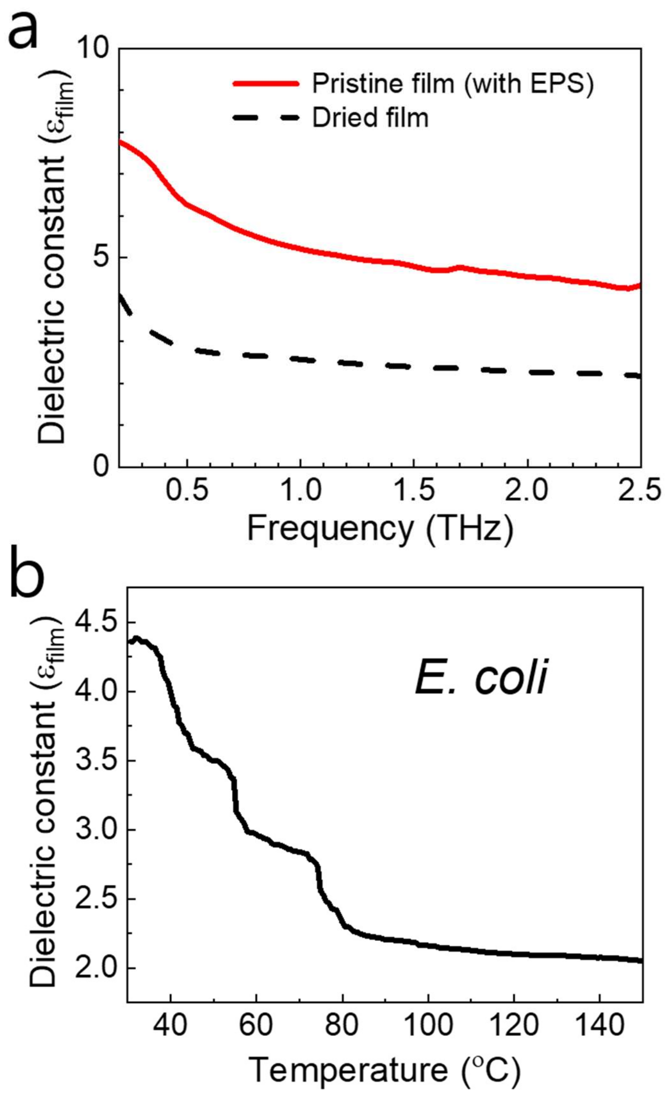

Supplementary Figure 3 a Dielectric constant of *E. coli* film for the pristine (red) and dried conditions (black). We obtained the dielectric information by fabricating a thick pallet with the thickness of 167 μm. The pristine film contains extracellular polymer substances (EPS) whose composition is mostly water and contains biological substances. **b** Dielectric constant of *E. coli* film with a thickness of 30 μm as a function of temperature for the data shown in Fig. 2**e** of the main text. The dielectric constant was obtained from the frequency shift (Δf) and the explicit relation shown in Supplementary Fig. 2.

S4. Microscopic image of the cells

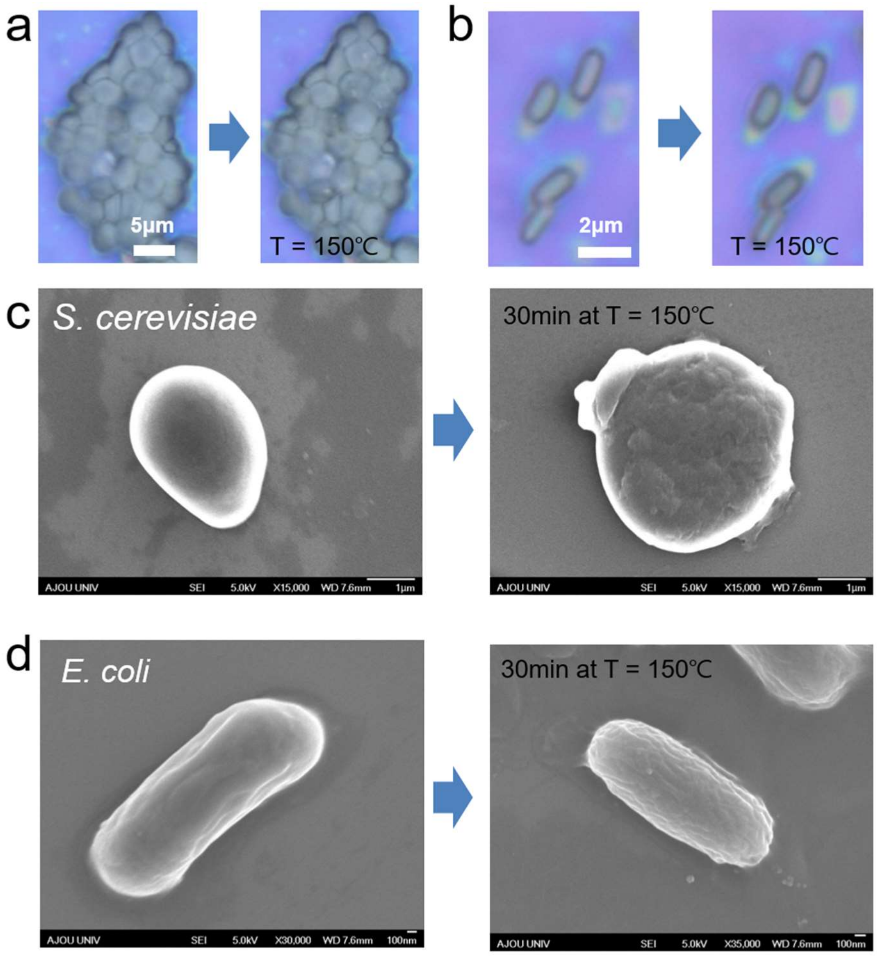

Supplementary Figure 4 a Representative optical microscopy images for individual yeasts before (left) and after (right) the heating (for 30 mins at T = 150 °C). **b** Microscopic images of *E. coli* before (left) and after (right) the heating. **c** Scanning electron microscopy (SEM) images of yeasts before and after the heating. **d** SEM images of *E. coli* before and after the heating. There is no noticeable heat-induced change with respect to the optical microscope images. Contrarily, through SEM images, we can clearly identify the nanoscale wrinkles in the cell walls after the heating for both yeast and *E. coli*. In other words, although they preserve their overall shape, they exhibited the morphology change resulting in the THz dielectric constant change.

S5. Thermal gravimetric analysis on bacterial films

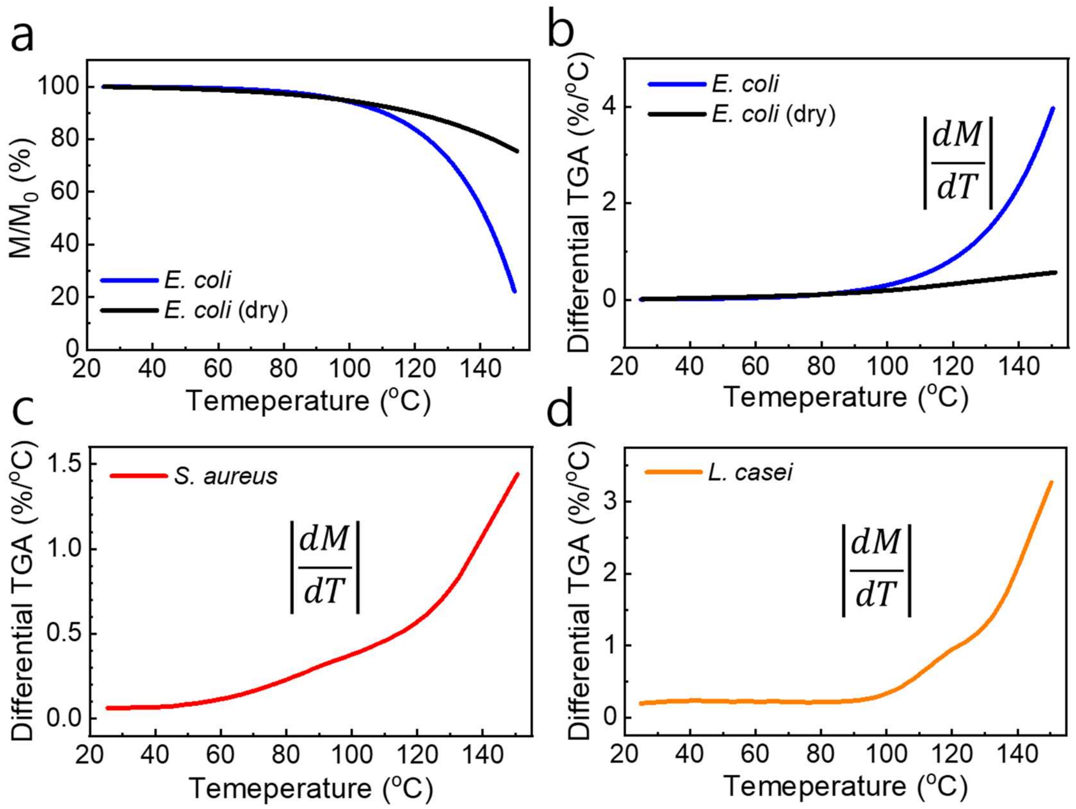

Supplementary Figure 5 a Thermal gravimetric analysis (TGA) on *E. coli* films for both as-prepared (blue) and dried (black) films. We increased the substrate temperature from 25 °C to 150 °C while monitoring their mass changes. For both films, the microbial mass decreased by about 8% at 100 °C. Conversely, at the higher temperature (T > 100 °C) the mass change is more significant for the as-prepared films with only 20% left at 150 °C, which is owing to the water evaporation. The water evaporates gradually without exhibiting abrupt change at the specific temperature especially until it reached 100 °C. **b** Differential TGA results (extracted from **a**), indicating that the temperature-dependent mass analysis cannot provide information useful for the identification of individual bacteria. **c** Differential TGA for *S. aureus* (red). **d** Differential TGA for *L. casei* (orange) films. Again, we could not identify peaks that are useful for the identification of individual pathogens.

S6. Dynamical change of microbial film at fixed temperature

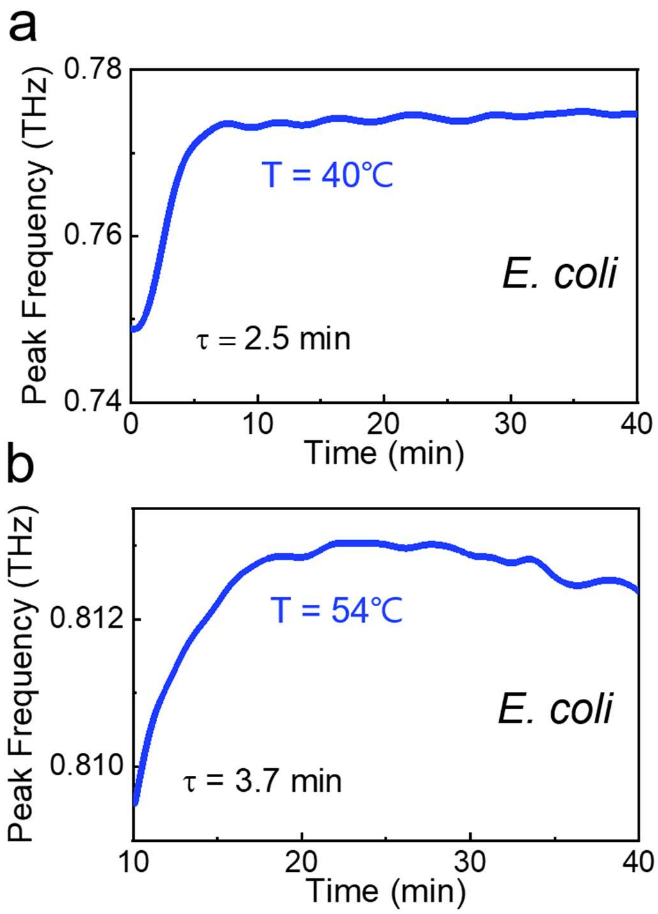

Supplementary Figure 6 A series of differential thermal curves of *E. coli* at the fixed annealing temperatures for **a** growth (40 °C) and **b** inactivation (54 °C) temperatures. For the inactivation condition, we increased the temperature gradually (3 °C/min) until it reached T = 54 °C at 10 min to avoid a delamination effect. The dielectric constant increases with time constants of 2.5 min and 3.7 min, respectively for the growth and inactivation conditions. Here, we fitted the curves with the relation of $\Delta f = \Delta f_{\mathrm{sat}}(1-\exp(-t/\tau))$, where Δf_{sat} is the maximum frequency shift and τ is the time constant. Therefore, monitoring of dielectric constant using an in-situ THz spectroscopy with metasensors will be effective for interrogating their dynamical phases under various microbial procedures. We note that this transient behavior can limit the measurement time, whereas we could improve the detection speed with more precise control over the environmental and experimental conditions.

S7. Differential thermal curves for other microbial species

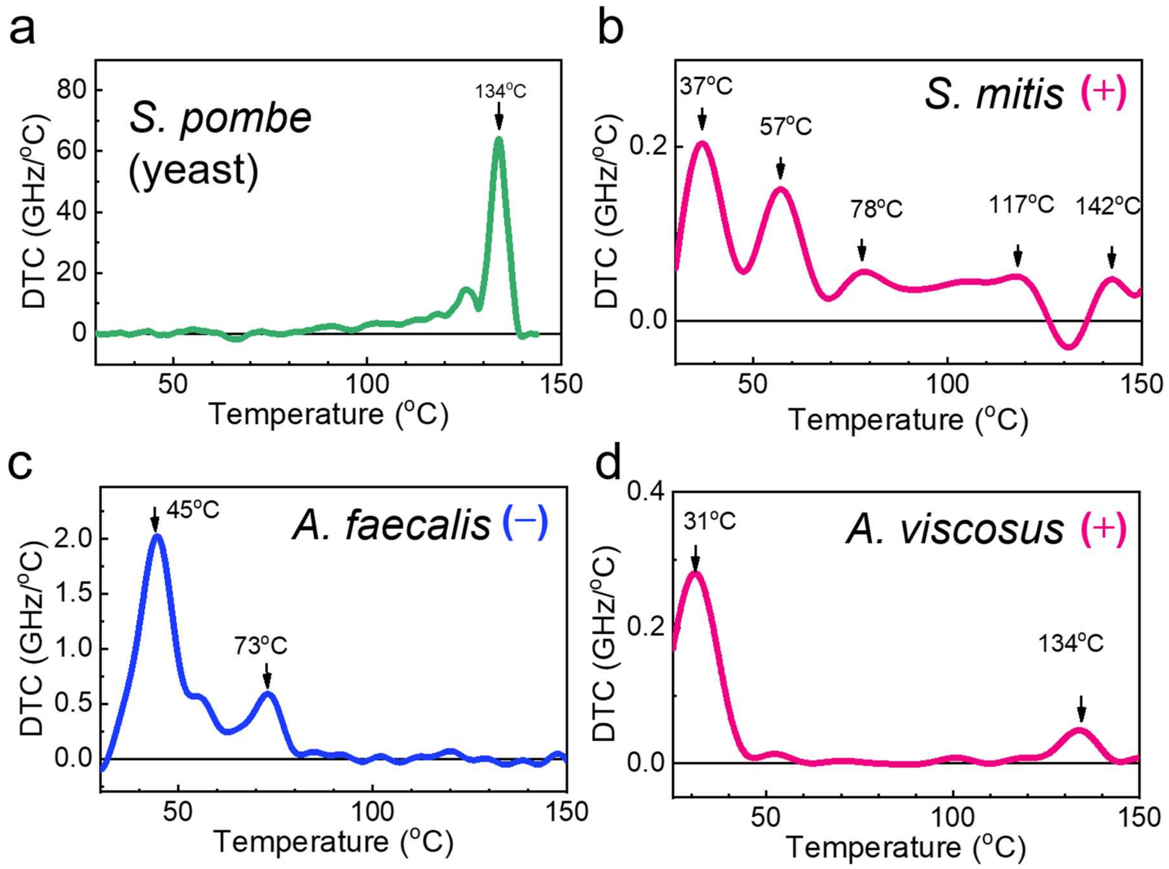

Supplementary Figure 7 Differential thermal curves for four different species of yeast and bacterial layers with **a** *S. pombe*, **b** *S. mitis*, **c** *A. faecalis*, and **d** *A. viscosus*. *S. pombe* is used in traditional brewing and also plays as a model microorganism in molecular and cell biology[3]. *S. mitis* inhabits the human mouth and throat and could cause bacterial endocarditis[4]. *A. faecalis* causes unitary tract infection[5]. *A. viscosus* causes periodontal disease in animals and has also known to cause endocarditis[6]. Positive (+) and negative (–) sings denote the gram-positive and gram-negative bacteria, respectively.

S8. DTC peak amplitudes and positions with error-bars

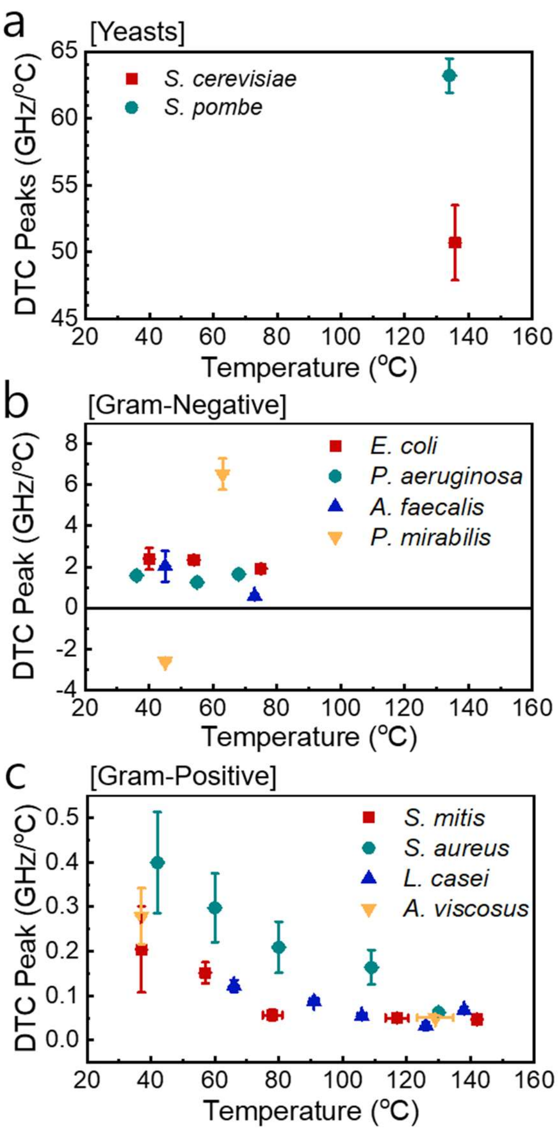

Supplementary Figure 8 Unnormalized peak amplitudes for the representative data shown in Fig. 4 of the main text and Supplementary Fig. 7 for **a** yeasts, **b** gram-negative bacteria, and **c** gram-positive bacteria. Data are presented as mean values, whereas the error bars represent the standard deviations obtained during the fitting process. The DTC amplitudes show relatively large errors, whereas it is the DTC peak position which is useful for the selective identification. The DTC amplitude could vary depending on the amount of the microbes, the variations in the temperature increase rate, and potentially, the water constituents in the microbes.

S9. DTC for microbial films grown at different conditions

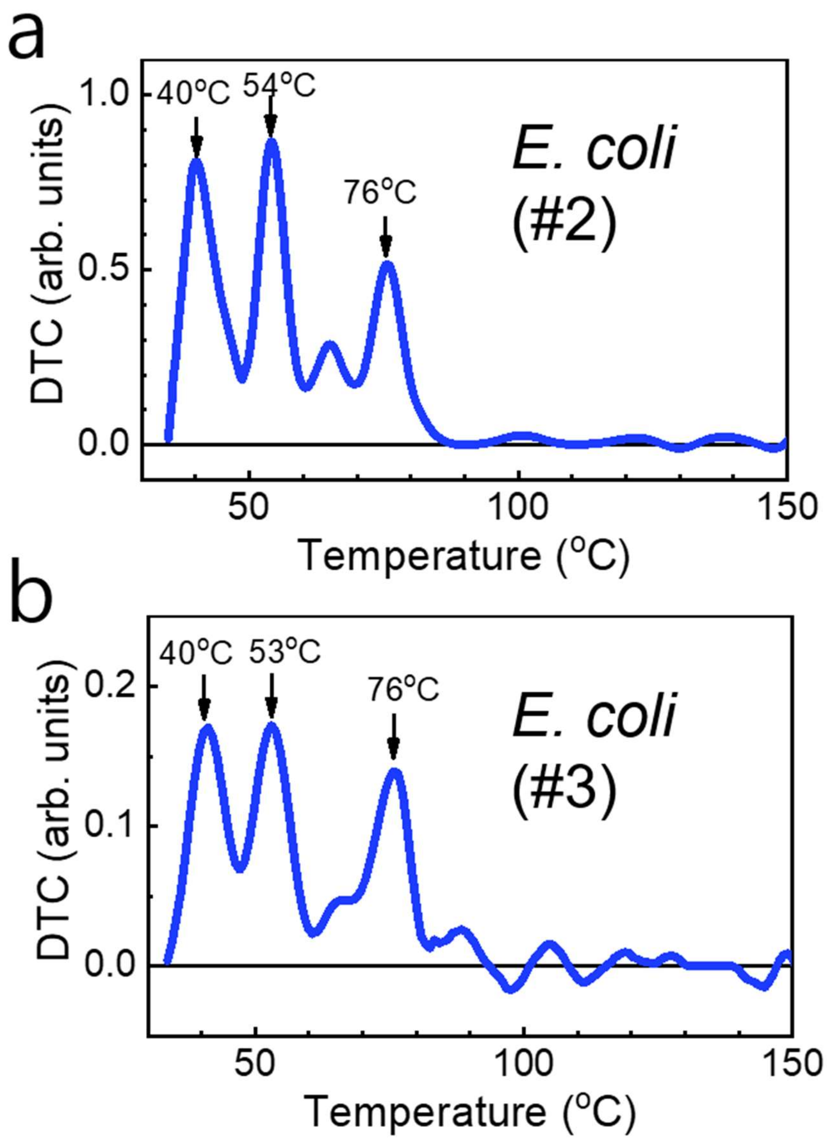

Supplementary Figure 9 A series of differential thermal curves of *E. coli* layers grown on different days, which is comparable to that of Fig. 2f of the main text. DTCs have a similar shape with distinct peaks corresponding to the growth, inactivation, and DNA denaturation. Conversely, there are slight variations in terms of the position, amplitude, and width of the peaks. We note that it inevitable that the growth condition will vary from sample to sample due to the small variation of temperature, humidity, and the ion density of culture medium[7]. Similar behavior was found for other bacterial species.

S10. DTC results for thick films

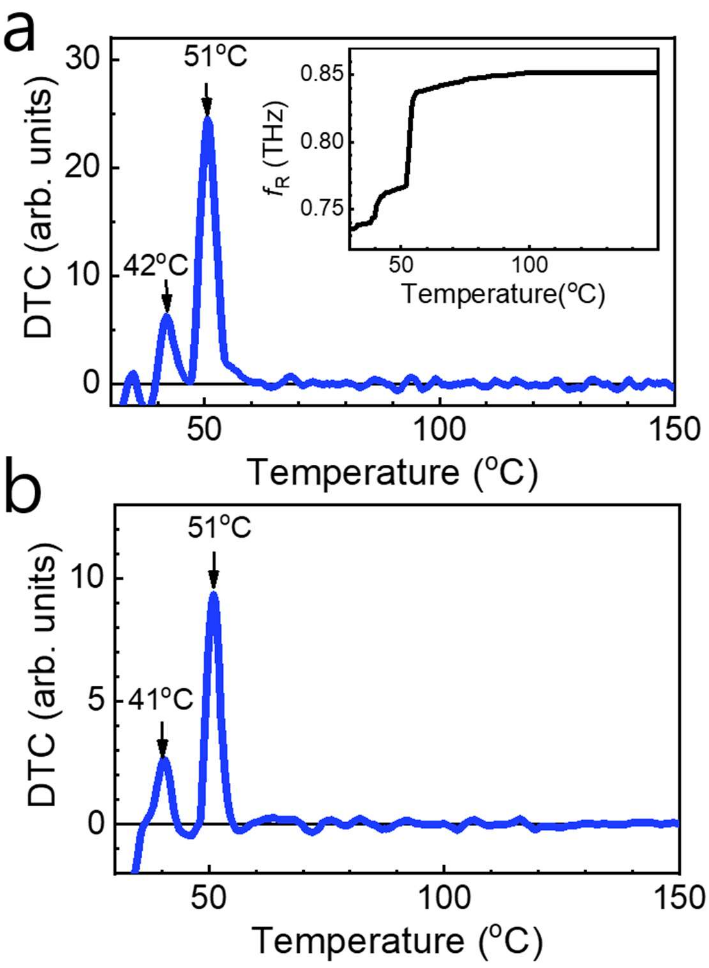

Supplementary Figure 10 Differential thermal curves measured for thick *E. coli* films for a series of *E. coli* grown on different days. The thickness of the films reached 300 μm, whereas most of microbial films used in other experiments were 20 – 30 μm. Interestingly, we found that only two peaks appeared in the case of the thick samples, i.e., for the growth and inactivation temperatures; this necessitates future investigation.

S11. DTC results using functionalized metasensors

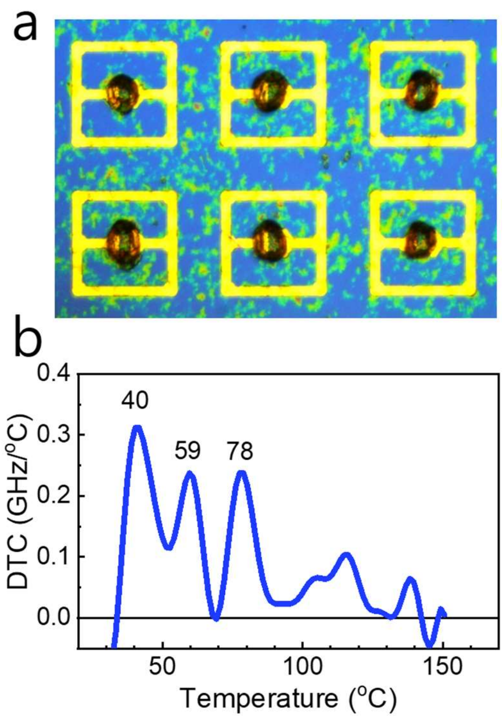

Supplementary Figure 11 We performed DTC experiments when the bacterial layers were confined in the gap area (3×10 μm^2), reducing the amount of pathogens dramatically. We fabricated the functionalized metasensors according to the literature[8]; in other words, we filled the gap area with the adhesive polymers, whereas pathogens are repelled outside the gap area. As shown in the microscopic image in **a**, the *E. coli* layer was transferred from the culture medium using a swab, followed by rinsing process. The thickness of the *E. coli* layer confined in the gap region was around 2 μm; therefore, the amount of the microbes reached 0.2% of the microbial layers considering the reduced coating area. The DTC results are shown in **b**, which is consistent with those of the relatively thick microbial films, whereas the reduced DTC amplitudes are due to the reduced bacterial layer thickness. Our results can be extended to improve the feasibility of on-site applications by using the squeegee tools or by incorporating the fluidic devices combined with the electrophoresis technique for sweeping the pathogens into the gap region efficiently[3,9].

Supplementary Table 1 Peak temperature values from the independent DTC measurements in terms of mean and standard deviation (in parentheses). The sample size (*n*) used for statistics was *n* = 6 for *E. coli*, *n* = 4 for *S. aureus* and *L. casei*, and *n* = 3 for the rest of bacteria (unit: °C).

Microorganism		Growth	Thermal inactivation	DNA denaturation	Cell wall destruction
Yeast	*S. cerevisiae*				137 (1.1)[10]
	S. pombe	-	-	-	134 (0.9)[10]
Bacterium (gram-negative)	*E. coli*	40.7 (0.7)[11]	53.9 (3.3)[11]	76.1 (1.1)[7]	-
	P. aeruginosa	35.7 (4.2)[12]	55.1 (0.2)[13]	67.9 (1.6)[14]	-
	P. mirabilis	45.3 (0.9)[15]	63.1 (4.9)	-	-
	A. faecalis	44.9 (0.5)	-	72.8 (1.0)	-
Bacterium (gram-positive)	*S. aureus*	43.7 (0.7)[16]	63.6 (3.2)[17]	81.8 (2.7)[18]	106 (4.5), 128 (4.0)
	L. casei	-	65.7 (1.0)[19]	90.6 (2.0)	106 (1.5), 126 (2.8), 139 (2.5)
	S. mitis	36.6 (0.4)[20]	55.5 (2.2)	78.0 (0.9)	115 (2.5), 143 (1.3)
	A. viscosus	31.2 (1.0)[21]	-	-	132.6 (3.2)

References

1. Park S, Yoon S, Ahn Y. Dielectric constant measurements of thin films and liquids using terahertz metamaterials. *RSC advances* **6**, 69381-69386 (2016).
2. Park DJ, Park S, Park I, Ahn Y. Dielectric substrate effect on the metamaterial resonances in terahertz frequency range. *Current Applied Physics* **14**, 570-574 (2014).
3. Yu ES*, et al.* Nanoscale terahertz monitoring on multiphase dynamic assembly of nanoparticles under aqueous environment. *Advanced Science* **8**, 2004826 (2021).
4. Pearce C*, et al.* Identification of pioneer viridans streptococci in the oral cavity of human neonates. *Journal of medical microbiology* **42**, 67-72 (1995).
5. Huang C. Extensively drug-resistant Alcaligenes faecalis infection. *BMC infectious*

diseases **20**, 1-11 (2020).

6. Eng RH, Corrado ML, Cleri D, Cherubin C, Goldstein EJ. Infections caused by Actinomyces viscosus. *American journal of clinical pathology* **75**, 113-116 (1981).
7. Khandelwal G, Bhyravabhotla J. A phenomenological model for predicting melting temperatures of DNA sequences. *PloS one* **5**, e12433 (2010).
8. Cha SH, Park SJ, Ahn YH. Investigation of Sensitivity Distribution in THz Metamaterials Using Surface Functionalization. *Current Optics and Photonics* **3**, 566-570 (2019).
9. Ryu Y-S, Lee D-K, Kang J-H, Lee S-H, Yu E-S, Seo M. Ultrasensitive terahertz sensing of gold nanoparticles inside nano slot antennas. *Optics Express* **25**, 30591-30597 (2017).
10. Xu S, Xu X, Zhang L. Effect of heating on chain conformation of branched β-glucan in water. *The Journal of Physical Chemistry B* **117**, 8370-8377 (2013).
11. Guyot S*, et al.* Extremely rapid acclimation of Escherichia coli to high temperature over a few generations of a fed-batch culture during slow warming. *Microbiologyopen* **3**, 52-63 (2014).
12. Chandran M, Duraipandi V, Yuvaraj D, Vivek P, Parthasarathy N. Production and extraction of bacterial pigments from novel strains and their applications. *Res J of Pharma Biological and Chem Scie* **5**, 584-593 (2014).
13. O'Toole A, Ricker EB, Nuxoll E. Thermal mitigation of Pseudomonas aeruginosa biofilms. *Biofouling* **31**, 665-675 (2015).
14. Tahmasebi H, Dehbashi S, Arabestani MR. High resolution melting curve analysis method for detecting of carbapenemases producing pseudomonas aeruginosa. *J Krishna Inst Med Sci Univ* **7**, 70-77 (2018).
15. Tan H, Wang R, Zhang H, Nie L, Yao S. Detection and analysis of the temperature-

dependent growth characteristics of Proteus mirabilis using a bulk acoustic wave ammonia sensor. *Bioelectrochemistry and bioenergetics* **44**, 83-88 (1997).

16. Akram M, Shafaat S, Bukhari DA, Rehman A. Characterization of a thermostable alkaline protease from Staphylococcus aureus S-2 isolated from chicken waste. *Pakistan J Zool* **46**, 1125-1132 (2014).
17. Shafiei Y, Razavilar V, Javadi A. Thermal death time of staphylococcus aureus (PTCC= 29213) and Staphylococcus epidermidis (PTCC= 1435) in distilled water. *Aust J Basic Appl Sci* **5**, 1551-1554 (2011).
18. Hsiao P-K, Chen W-T, Chang K-C, Ke Y-J, Kuo C-L, Tseng C-C. Performance of CHROMagar Staph aureus and CHROMagar MRSA for detection of airborne methicillin-resistant and methicillin-sensitive Staphylococcus aureus. *Aerosol Science and Technology* **46**, 297-308 (2012).
19. Liu X, Champagne CP, Lee BH, Boye JI, Casgrain M. Thermostability of probiotics and their α-galactosidases and the potential for bean products. *Biotechnology research international* **2014**, 472723 (2014).
20. Rukke H, Hegna I, Petersen F. Identification of a functional capsule locus in Streptococcus mitis. *Molecular oral microbiology* **27**, 95-108 (2012).
21. Könönen E, Wade WG. Actinomyces and related organisms in human infections. *Clinical microbiology reviews* **28**, 419-442 (2015).
